# Special Issue: Molecular Ecology, Physiology and Biochemistry of Insects 3.0

**DOI:** 10.3390/ijms25105390

**Published:** 2024-05-15

**Authors:** Klaus H. Hoffmann

**Affiliations:** Animal Ecology I, University of Bayreuth, University Street 30, 95448 Bayreuth, Germany; klaus.hoffmann@uni-bayreuth.de

With the outstanding work of Sir Vincent B. Wigglesworth and his book titled *Insect Physiology* [1], which was published in 1934, started a period of rapid development in “classical” insect physiology and biochemistry. Today, many biochemical and physiological processes in insects serve as models for corresponding processes in vertebrates and humans. With around 10 million estimated species, insects represent the most diverse group of animals on Earth. Their extreme adaptation abilities allow them to live from the Arctic to the equator and from the seashore to the high mountains. Insects are also well represented in freshwater, but hardly in marine waters.

The revolutionary development of molecular biological methods over the recent 50 years and their application to insects has enabled novel and deeper insights into the biochemistry, physiology, and environmental adaptation processes of insects. Currently, several hundred scientific papers on molecular insect ecology, physiology, and biochemistry appear in up to 100 journals every year.

In 2019, we took this development as an opportunity to call for articles to be submitted for a first Special Issue in MDPI’s *International Journal of Molecular Sciences* (IJMS) on the molecular ecology, physiology, and biochemistry of insects, and 15 papers were published in 2019/2020. In the present third Special Issue on that topic, eight papers have been printed. A fourth edition is in progress with a deadline of 30 August 2024 for manuscript submission. Another eight articles were already published to date for the fourth edition.

The eight articles from “Molecular Ecology, Physiology and Biochemistry 3.0” comprise one review and seven original papers. The review by Sun et al. 2023 [Contribution 1] deals with a special way of life of insects, the so-called invasive species. Invasive species are not native to a particular area. They are introduced into a new region, often by accident, and can cause great economic and environmental damage. High-throughput sequencing technologies have been used to study the mechanisms through which insects achieve invasion. Sun et al. review recent whole genome sequencing-based advances in revealing important adaptation mechanisms of invasive insects. They also discuss whole-genome sequencing-based use of prevention and control technologies against invasive insects.

Two of the original papers deal with honeybees, *Apis mellifera*. Isani et al. (2023) [Contribution 2] present an SDS-polyacrylamide electrophorese/mass spectrometry-based assay of the honeybee hemolymph proteins, which can be used to measure quantitative changes in protein concentrations over the course of the year. Changes in these biomarkers may mirror physiological changes during the reproductive season of the honeybees in the field. The paper by Zhang et al. (2023) [Contribution 3] confirms that DNA methylation and histone modifications can establish distinct gene expression patterns, leading to caste differentiation. Their results suggest that H3K4me1 (histone H3 lysine 4 mono-methylation) modifications, both in queen and worker larvae, are linked to caste differentiation.

Jan Veenstra (2023) [Contribution 4] studied caste specifics in another eusocial group of insects, the termites. In well-established colonies of higher termites, the only food the queen receives is saliva from the workers. The queen can live for many years and produce up to 10,000 eggs. The saliva of the workers contains proteins resembling honeybee royal jelly. The major protein in the saliva was identified as a homolog of a cockroach allergen [2]. The protein is not expressed in soldiers, and, like the major royal jelly proteins in honeybees, it is expressed in young but not old workers. In contrast to the original cockroach allergen, the termite salivary paralog contains the essential amino acids methionine, cysteine, and tryptophan, thus allowing it to become more nutritionally balanced.

The two papers by Bloskie and Storey (2023) [Contribution 5] and Su et al. (2023) [Contribution 6] present molecular mechanisms underlying long-term evolutionary adaptations to exceptional environmental conditions. The golden gall fly, *Eurosta solidaginis*, is a well-studied model of insect freeze tolerance [3]. Larvae accept ice in the extracellular space while protecting the intracellular space with huge amounts of cryoprotectants, such as glycerol. The present studies provide evidence for an epigenetic-mediated transcription suppression in winter-diapausing larvae, especially via histone H3 and H4 modifications. The mountain butterfly, *Parnassius glacialis*, probably originated in the high-altitude Tibet plateau and later dispersed into low-altitude regions of central China. Su et al. obtained high-throughput RNA Seq data from twenty-four adult individuals in eight regions and firstly identified the diapause-linked gene expression pattern that likely correlates with local adaptations and provides insights into the evolution of diapause in this mountain butterfly species.

The fall armyworm, *Spodoptera frugiperda* (*Sfru*), is a worldwide general lepidopteran pest with remarkable adaptations to environment and stresses. Odorant binding proteins (OBPs) and chemosensory proteins (CSPs) play crucial roles in insect chemoreception. Jia et al. (2023) [Contribution 7] screened for genome-wide *SfruOBPs* and *SfruCSPs*, and analyzed their expression patterns across all development stages and sexes. A total of 33 *OBPs* and 22 *CSPs* were found. The majority of *SfruOBP* genes were most highly expressed in the adult females and males, while most *SfruCSPs* were highly expressed in larval and egg stages. Competitive binding studies with the widely expressed *SfruOBP31* revealed a broad functional-related binding to host plant odorants, sex pheromones, and insecticides.

Chagas disease is caused by the protozoan *Trypanosoma cruzi* and is spread mostly by insects of the subfamily Triatominae. Currently, 157 species of triatomines are known, and the generation of hybrids is common between the species. dos Reis et al. (2023) [Contribution 8] studied whether different karyotypes could act in the reproductive isolation of triatomines and found out that during their evolutionary process, at least nine cladogenetic events associated with alterations in the number of chromosomes may have occurred.

The papers within this Special Issue will certainly help to better understand how physiological and biochemical processes in insects are regulated and at what time. This can help to manipulate them, thereby providing new opportunities for practical application, for example, in an ecologically friendly insect pest control.
